# Genetics of Malaria Inflammatory Responses: A Pathogenesis Perspective

**DOI:** 10.3389/fimmu.2019.01771

**Published:** 2019-07-30

**Authors:** Carlos Penha-Gonçalves

**Affiliations:** Instituto Gulbenkian de Ciência, Oeiras, Portugal

**Keywords:** malaria, NOS2 gene, type 1 interferon receptor 1 (IFNAR1), genetics, TNFA gene, CD40LG gene, HMOX1 gene, TLRs (toll-like receptors)

## Abstract

Despite significant progress in combating malaria in recent years the burden of severe disease and death due to *Plasmodium* infections remains a global public health concern. Only a fraction of infected people develops severe clinical syndromes motivating a longstanding search for genetic determinants of malaria severity. Strong genetic effects have been repeatedly ascribed to mutations and allelic variants of proteins expressed in red blood cells but the role of inflammatory response genes in disease pathogenesis has been difficult to discern. We revisited genetic evidence provided by inflammatory response genes that have been repeatedly associated to malaria, namely TNF, NOS2, IFNAR1, HMOX1, TLRs, CD36, and CD40LG. This highlighted specific genetic variants having opposing roles in the development of distinct malaria clinical outcomes and unveiled diverse levels of genetic heterogeneity that shaped the complex association landscape of inflammatory response genes with malaria. However, scrutinizing genetic effects of individual variants corroborates a pathogenesis model where pro-inflammatory genetic variants acting in early infection stages contribute to resolve infection but at later stages confer increased vulnerability to severe organ dysfunction driven by tissue inflammation. Human genetics studies are an invaluable tool to find genes and molecular pathways involved in the inflammatory response to malaria but their precise roles in disease pathogenesis are still unexploited. Genome editing in malaria experimental models and novel genotyping-by-sequencing techniques are promising approaches to delineate the relevance of inflammatory response gene variants in the natural history of infection thereby will offer new rational angles on adjuvant therapeutics for prevention and clinical management of severe malaria.

## Introduction

**Figure 1:**
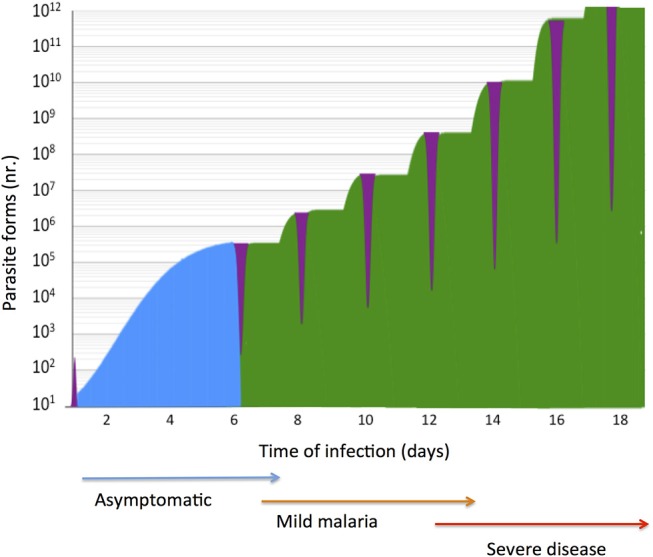
Natural history of malaria infection in a naïve susceptible host. *P. falciparum* expansion in the liver and in blood stage infection is represented in a logarithmic scale. Exposure of parasite extra-cellular forms (purple) to the host immune system is brief in case of sporozoites (<1 h) and momentaneous in the case of free merozoites in the blood (few minutes). In a typical infection liver stage parasites (blue) expand in few dozens of hepatocytes in circa 5.5 days, a clinically unapparent phase (blue arrow). Synchronous blood stage parasite expansion is represented (green), considering six rounds of trophozoite division during erythrocytic schizogony. In naïve individuals blood stage infection causes tertian fever (one brood infection) and mild malaria (brown arrow) but the clinical severity (red arrow) is co-determined by parasite pathogenicity factors and by the host genetic make-up.

During the last decade intensive efforts have succeeded in implementing malaria epidemiological control measures and in deploying new anti-malaria drugs that have significantly decreased the disease burden across the world (1). Nevertheless, malaria claims close to half a million deaths each year (2) demanding deeper understanding of severe malaria pathogenesis (3). Malaria is caused by mosquito-transmitted *Plasmodium* protozoan parasites that develop in multiple stages within the vertebrate host. The chronology of infection exposes the host to distinctive intracellular and extracellular forms that emerge sequentially during the natural history of infection (1, 4) (Figure 1). Free parasite forms- sporozoites and merozoites- are non-replicative and obligatory for life cycle progression. Although directly exposed to the immune system free parasites represent brief phases in infection that do not elicit protective responses in naive subjects (5). In contrast, intracellular parasite forms hidden in hepatocytes or in erythrocytes, undergo significant expansion, damaging, and ultimately destroying infected cells while offering a large number of inflammatory triggers and immunogenic targets (4, 6, 7).

Nevertheless, the effectiveness of anti-parasite responses is defied both by the dynamics of infection (e.g., high parasite growth rate) and by variations in the parasite antigenic complexity represented by stage-specific sets of antigens or by mechanisms of antigenic diversification and clonal antigenic variation (7–9). As a result the host immune system engages in multiple ineffective responses, a scenario that allows parasite cell-cycle progression and transmission to the next mosquito host. Clinical outcomes of blood-stage infection range from unapparent infection to life-threatening conditions depending on the caliber and effectiveness of anti-parasite responses (10). The severity of clinical manifestations result from direct parasite effects on infected cells (e.g., hemolysis), systemic effects of parasite activity and growth (e.g., malaria paroxysms and metabolic imbalances) as well as organ lesions (mainly in brain, lungs, liver, spleen, kidney, and placenta) generated by massive parasite clearance reactions and mal-adapted inflammatory responses (11). Thus, exacerbation of inflammatory responses during infection is a key determinant in development of immunopathology and organ dysfunction associated with severe malaria syndromes (12–14). This review scrutinizes genetic association evidence supporting that pro-inflammatory genetic variants have dual roles in disease pathogenesis.

## Anti-parasite Responses

The host response to malaria includes a wide range of both cell-intrinsic and systemic mechanisms but the initial responses in a naive host are mostly driven by non-specific reactions (1). Liver stage infection is clinically silent as the number of infected hepatocytes is relatively low (15). Liver infection progression is counteracted by hepatocyte killing mechanisms, including activation of hepatocyte-intrinsic apoptosis pathways (16–20) and innate responses of liver non-parenchymal cells (21–24). Nevertheless, completion of liver stage infection is usually secured by parasite-driven mechanisms that inhibit apoptosis of infected hepatocytes (19, 25), inhibit hepatocyte autophagy (26) or suppress liver macrophages responses (27, 28). As a result, a small number of infected hepatocytes sustain parasite growth and generate a relatively high number of merozoites that are freed in the bloodstream initiating blood stage infection.

An archetypal infection in a naive host leads to exponential increase blood stage parasites due to the cyclic expansion of asexual parasite forms (Figure 1) but in natural infection parasite growth is many times counteracted by strong host responses. Parasite molecules expressed in the surface of infected erythrocytes (IE) induce powerful innate responses leading to detection, engulfment, and destruction of large numbers of IE mainly in the spleen and liver (29–31). Pattern recognition receptors (e.g., TLRs) expressed by professional antigen presenting cells contribute to IE recognition and engulfment (32, 33). Phagocytosis triggers a respiratory burst response involving reactive oxygen species in intracellular parasite killing (34) but also leads to production of cytokines and chemokines that engage and polarize subsequent antibody- and cytotoxicity-mediated adaptive immune responses (35).

In this pro-inflammatory environment the mounting of adaptive responses in secondary lymphoid organs, mainly in the spleen white pulp (31) favors the generation of cytophilic or opsonizing antibodies that recognize *Plasmodium* antigens expressed in the surface of infected erythrocytes (36). Similarly, opsonizing antibody responses target free merozoites for destruction and correlate with disease protection (37, 38) and infection resolution (39–41). Nevertheless, variation and diversification of parasite antigens expressed in the erythrocyte surface subvert adaptive responses thereby sustaining infection progression (42). Furthermore, parasite virulence factors such as higher intrinsic growth rate, broad erythroid tropism, and cyto-adherence (43–46) are determinants of increased blood-stage parasite loads that in turn exacerbate the pro-inflammatory responses associated with severe disease (10, 47).

## Immunopathology and Severe Outcomes

Several immunologic mechanisms triggered by infection in non-immune individuals paradoxically favor disease development as they reveal inefficiencies in eliminating the parasite. The host immune system reacts to parasite molecules released during IE rupture (e.g., hemozoin pigment and lipid-associated GPI anchors, so-called “malaria toxins”) through innate immunity receptor signaling (e.g., TLRs) leading to release of pro-inflammatory mediators, namely TNF-alpha, IL-1β, IL-6, and IFN-gamma (48–50). In overt blood-stage infection this inflammatory environment is amplified by reciprocal activation loops involving monocytes, NKT cells, T cells, and endothelial cells (11). Systemic microvascular endothelial activation by inflammatory mediators, IE and parasite components (e.g., nucleosomes and microvesicles) plays a decisive role in organ inflammation (51, 52) with marked up-regulation of adhesion molecules (e.g., ICAM1) and imbalances in vasoactive mediators (angiotensins, endothelin-1, and nitric oxide) (53–55). Often, adhesion of activated monocytes, T cells and platelets (56–58) leads to altered vascular permeability and coagulation disturbances with induction of endothelial cell damage and death. In counterbalance, tissue damage cues such as alarmins and cytokines released by parenchymal cells engage macrophages and lymphoid cells in inflammation resolution and tissue repairing responses (59). These general tissue protective responses that counteract inflammation may operate in malaria by induction of regulatory T cells (60, 61), production of anti-inflammatory cytokines [e.g., TGF-β (62, 63)] and stimulation of oxidative-stress protection systems [e.g., heme oxigenase 1 and nitric oxide (59)]. The inflammatory mechanisms and tissue protective responses elicited by infection interplay with parasite factors and microanatomy components in different organs to determine tissue-specific vulnerability to immunopathology (64), ultimately allowing development of specific malaria clinical syndromes.

## Inflammatory Response Genes and Malaria Clinical Outcomes

The epidemiology of severe malaria suggests that cumulative exposure to the malaria parasite confers resilience against life-threatening disease (1). Nevertheless, malaria epidemiology alone does not explain why only a relatively small fraction of primary *Plasmodium* infections develop severe clinical forms. Compelling genetic evidence collected in recent years shows that malaria contributed to shape the human genome and it is established that variants in genes coding for proteins expressed in erythrocytes are major genetic determinants of resistance to infection and resilience to severe disease (65–71).

Interestingly, a growing number of genetic association studies also report that inflammation-related genes have a role in determining the course and clinical outcomes of infection. Yet, the action of individual genetic variants has been difficult to resolve and in some cases genetic variants appear to have pleiotropic effects (72). This is in part due to disparities in study design, regional differences in intensity of malaria transmission, diversity of *Plasmodium* biotypes, and relatively small sample sizes with unknown genetic substructure, particularly in Africa (70, 73). Usage of larger sample sizes in multicentric studies do not overcome the possibility that functional consequences of a given genetic variant may vary among geographic locations (74). Moreover, the diversity of malaria phenotypes and *Plasmodium* species used in case definition of different studies may preclude the identification of genetic variants as general malaria risk factors. Nevertheless, cumulative evidence corroborating the association of specific inflammatory genes with malaria phenotypes is instrumental to value their contribution to malaria pathogenesis and to scrutinize their roles during infection.

**Figure 2:**
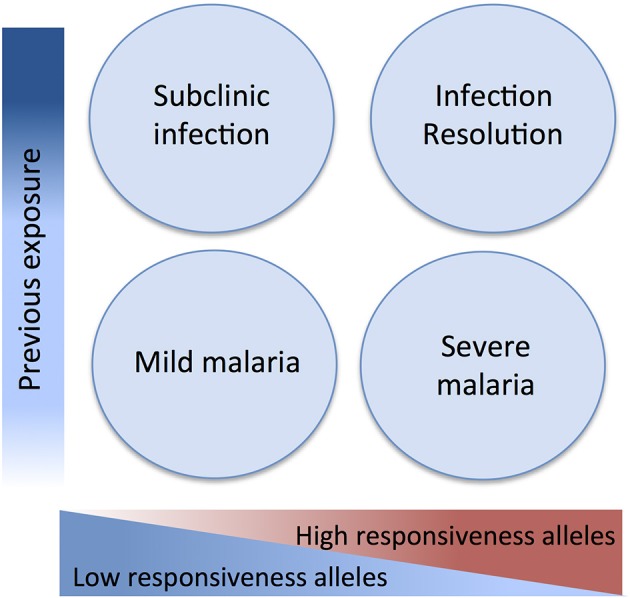
Genetic variants in inflammatory response genes and infection outcomes. Genetic variants controlling inflammatory responsiveness are proposed determinants of malaria clinical outcomes dependent on history of exposure to infection. Under the framework of a polygenic model of malaria susceptibility the additive effects of genetic variants conferring strong inflammatory responsiveness to infection concur to severe disease but may favor efficiency of anti-parasite responses in repeatedly exposed individuals. On the other hand, joint effects of alleles conferring low inflammatory responsiveness results in increased resilience to severe malaria but are associated to asymptomatic parasitemia in exposed individuals possibly due to inefficient parasite clearance.

Here we attempted to integrate evidence obtained in genetic association studies in light of a malaria pathogenesis model informed by experimental and epidemiological evidence. This model attributes a dual role to inflammatory responsiveness during the natural history of infection and takes in consideration the intra-host infection dynamics and the history of exposure to malaria (Figure 2). Accordingly, pro-inflammatory alleles promoting strong anti-parasite responses are predicted to increase resistance against infection and uncomplicated disease but also contribute to mal-adapted inflammatory responses that underlie progression to severe malaria syndromes. Conversely, alleles that mediate low inflammatory responsiveness may increase susceptibility to unapparent infection and favor mild malaria but would confer resilience to inflammation-driven severe organ damage. In this context, low inflammatory responsiveness offers an explanation for why individuals surviving initial infections resisting to severe malaria are inefficient in clearing the parasite and show long periods of asymptomatic parasitemia. This implies, that genetic control of malaria clinical outcomes afforded by genetic variance in inflammatory genes is modified by cumulative exposure to infection translating into efficient infection resolution or asymptomatic infection (Figure 2).

We followed a gene-centered approach to screen malaria association studies encompassing different clinical phenotypes, geographic regions, and *Plasmodium* species aiming to identify gene variants that have repeatedly produced evidence for positive association, namely in TNF, NOS2, IFNAR1, HMOX1, TLRs, CD36, and CD40LG genes (Supplementary Table 1). Subsequently, genetic effects of these individual variants were scrutinized across different case-control studies using a terminology that takes in account the malaria status of the groups of individuals that are compared in each study (Table 1). The analysis of the malaria association landscape in individual genes reconciled apparently incongruent data and offers a perspective on the roles of specific inflammatory mediators in malaria pathogenesis.

**Table 1:** Terminology of malaria genetic effects.

**Genetic effects in malaria case -control studies**
**Disease/infection susceptibility**
Increased risk of a malaria phenotype (or infection) when comparing to healthy (or uninfected) individuals.
**Disease/infection resistance**
Decreased risk of a malaria phenotype (or infection) when comparing to healthy (or uninfected) individuals.
**Disease progression**
Increased risk of a malaria phenotype when comparing with infected individuals (including asymptomatic parasite carriers).
**Disease protection**
Decreased risk of a malaria phenotype when comparing with infected individuals (including asymptomatic parasite carriers).
**Disease vulnerability**
Increased risk of a specific disease syndrome/outcome when comparing to other symptomatic clinical forms.
**Disease resilience**
Decreased risk of a specific disease syndrome/outcome when comparing to other symptomatic clinical forms.

*Genetic effects of alleles identified in association studies are denoted according the clinical status of the control group*.

## Tumor Necrosis Factor

Tumor necrosis factor (TNF) is a potent pro-inflammatory mediator involved in various steps of immune responses. It has long been recognized that TNF takes part in anti-*Plasmodium* responses that lead to intra-erythrocytic parasite killing and parasitemia reduction (75). Yet, TNF was also implicated as a causative factor in development of malaria-associated organ pathology (76–78). Elevation of TNF serum levels is a common finding in malaria episodes (79) and was noted that high TNF production capacity correlates with faster parasite clearance and with resolution of malaria attacks (80). The TNF response appears to follow the parasitemia kinetics with increased serum levels in children with high-density parasitemia and decaying with parasite clearance (81, 82). Nevertheless, TNF serum levels were repeatedly found increased in children with severe malaria (82–87) implying an intricate role in malaria pathogenesis (88).

**Figure 3:**
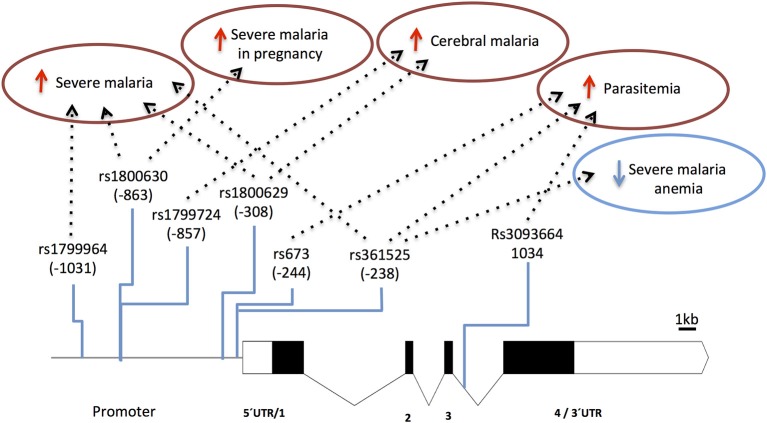
Genetic effects of TNF SNPs associated to clinical malaria. The scaled diagram of exon-intron structure of TNF gene shows malaria associated SNPs in the promoter region and one SNP in intron 3 (bar 1 Kb). Detrimental effects in malaria severity and parasitemia are represented by red arrows while beneficial effects are represented by blue arrows. TNF alleles associated with malaria are conferring several severe phenotypes with the exception of rs361525 that concomitantly is associated to protection from severe malaria anemia. For allele description and reported genetic effects (Supplementary Table 1).

Several TNF-α promoter single nucleotide polymorphisms (SNPs) that control gene expression or TNF production (89–92) are associated with control of parasitemia levels (92–95) and with increased anti-*P. falciparum* IgG levels (96). This suggests that TNF variants may play a role in the effectiveness of anti-parasite responses. Furthermore, the role of promoter variants in clinical malaria (e.g., rs1800629) is highlighted by reports associating TNF with vulnerability to severe disease, including cerebral malaria (97–102) and malaria in pregnancy (103, 104) (Figure 3 and Supplementary Table 1). Likely, control of TNF gene expression by these polymorphisms is context-dependent regarding secreting cell types, cell activation status, and action of other inflammatory mediators (105). Accordingly, it was reported that TNF2 allele (-308, aka rs1800629) controlled elevation of serum TNF in severe malaria patients but not in patients with uncomplicated malaria suggesting that its capacity to control gene expression may be modified in the course of infection (106). Also, TNF-238 G allele (rs361525) shows opposing effects on vulnerability to cerebral malaria and severe malaria anemia (107) suggesting differential roles of TNF in the spectrum of severe malaria syndromes. It should be noted that some of these association signals could result from genotypic combinations with neighboring genes, namely lymphotoxin-alpha (LTA) (88) and more detailed analysis of this region is needed to discern the involvement of TNF in human severe malaria (108).

Nevertheless, the data collectively suggest that TNF response capacity in malaria is controlled by TNF polymorphisms and that TNF represent a prototype of a pro-inflammatory factor in the natural course of infection; controlling parasite expansion in early stages of infection but acting in a context-dependent manner to increase the risk of specific severe malaria syndromes. The exact mechanisms by which TNF is involved in severe malaria remains unknown and requires a detailed functional testing of the network of TNF-TNFR family (88). However, it is noteworthy that other genetic modifiers of malaria susceptibility, namely NOS2, act as downstream effectors of TNF signaling encouraging further investigations on the intertwining of TNF and nitric oxide in disease development.

## Nitric Oxide Synthase 2 (NOS2)

Nitric oxide (NO) is a free radical involved in multiple biological processes, including inflammatory responses. Decreased NO bioavailability in severe malaria patients is a well-established pathogenesis factor (53, 109, 110) possibly impacting on endothelial activation (111) and macrophage polarization (112). Experimental mouse models of cerebral malaria demonstrated that increasing nitric oxide bioavailability protects against cerebral malaria by deterring systemic inflammation and the activation of immunocytes and endothelial cells (113–115). However, the therapeutic value of NO in severe malaria still lacks solid supportive evidence (116).

**Figure 4:**
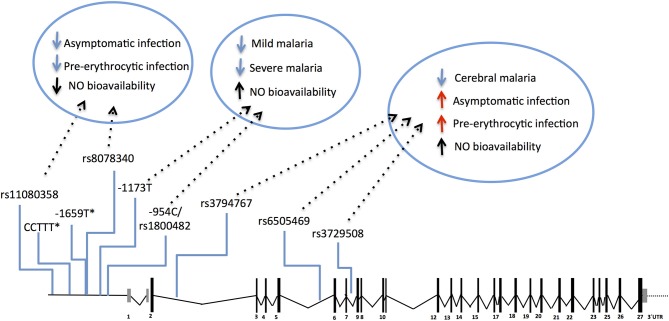
Genetic effects of NOS2 SNPs associated to malaria and nitric oxide bioavailability. The scaled diagram of NOS2 exon-intron structure depicts SNPs in the 5′region (rs11080358–rs1800482) and in the cistronic region (rs3794767–rs3729508) associated with malaria and with control of nitric oxide (NO) bioavailability. Genetic variants that decrease NO bioavailability in the 5′region have beneficial effects (blue arrows) in early stages of infection, possibly by favoring anti-parasite responses. In contrast, SNPs that increase NO bioavailability (mapping in the 5′ region or in the cistronic region) show an anti-inflammatory action favoring infection when parasite burden is low or when infection is unapparent (red arrows) but protect from mild malaria and increase resilience against severe malaria (blue arrows). (^*^) CCTTT and−1659 polymorphisms showed association with malaria severity and cerebral malaria but their role on NO bioavailability is not known. For allele description and reported genetic effects (Supplementary Table 1).

The NOS2 gene codes for the inducible NO synthase (iNOS) and has been probed in several genetic studies that collectively uncovered common genetic variants functionally correlated with increased NO bioavailability in infected individuals (117–119). Meaningfully, these variants have beneficial effects in clinical malaria, namely lower incidence of symptomatic re-infection (119) and uncomplicated malaria (117, 118, 120–123), decreased risk of severe malaria (121, 124, 125), including severe anemia and cerebral malaria (117, 118). Nonetheless, the same NOS2 alleles (e.g., rs6505469) were also associated with higher incidence of asymptomatic infection among apparently healthy individuals (117) (Figure 4 and Supplementary Table 1). This suggests that increased NO bioavailability in infected individuals counteracts detrimental effects of malaria inflammatory responses but is also impairing parasite clearance responses in early stages of infection. In contrast, other NOS2 variants in the promoter region that favor reduced NO bioavailability in absence of infection (e.g., rs8078340) were underrepresented in individuals with lower markers of exposure to pre-erythrocytic infection (e.g., anti-CSP antibodies) suggesting they contribute to resistance to infection (117).

The protective effects of NOS2 variants have not been consistently observed in different studies possibly owing to differences in the genetic variants analyzed, in the ethnic groups studied or in regional epidemiologic patterns of clinical malaria (126, 127). Nevertheless, the available genetic evidence cohesively supports the hypothesis that NO bioavailability holds an anti-inflammatory role in malaria pathogenesis that translates into decreased ability to clear the parasite in early stages of infection but enhancing protection against clinical inflammatory manifestations. The pathophysiological impact of NOS2 genetic variance is still not resolved but it should be noted that the NOS2 polymorphisms controlling NO bioavailability in absence of clinical malaria (e.g. rs8078340) are distinct from those associated with alterations of NO bioavailability in overt clinical disease (e.g. rs1800482) (117) (Figure 4). This implies that a distinct regimen of NOS2 genetic control is operating in infected individuals that should be taken into account when comparing groups of infected and non-infected individuals.

## Type 1 Interferon Receptor 1 (IFNAR1)

Type 1 interferons (IFN-1) are secreted by immune and non-immune cells including lymphocytes, macrophages, dendritic cells, fibroblasts, and endothelial cells. IFN-1 production is induced upon innate recognition of exogenous nucleic acids and proteins by innate receptors such as membrane-bound Toll-like receptors (TLRs) and cytosolic RNA helicases. Studies *in vitro* and in mice converge at the notion that sensing malaria parasites via innate immune receptors is linked to a IFN-1 response (128–130). A multi-level role of IFN-I signaling during *Plasmodium* infection in experimental models has been uncovered by the use of mice deficient in *Ifnar1* (a subunit of the IFN-1 receptor) or in components of the IFN-I induction pathways (e.g., *MDA5, MAVs, cGAS, STING, Irf7*, and *Irf3)* (129–131). Mouse studies identified distinct mechanisms that recruit the IFN-1/IFNAR1 axis to the response against malaria, including: (i) stimulation of hepatocyte-intrinsic interferon responses that counteract liver stage parasite expansion (22, 24); (ii) exacerbated IFN-1 production by plasmocytoid dendritic cells (DCs) leading to enhanced IFNAR1 signaling in conventional DCs that in turn drive responses against blood-stage parasite (131); (iii) IFNAR1-mediated effects in the CD4T helper-antibody axis that dampen the response against blood stage parasite allowing hyperparasitemia development (132–135); and (iv) the requirement for IFNAR1 expression in CD8T cells to license the cytotoxic effector functions that lead to cerebral malaria development in mice (136, 137).

Together these findings raise the possibility that IFN-1/IFNAR1 signaling exerts beneficial effects in early stages of infection when innate immune responses dominate. In contrast, detrimental effects are expected at later stages if IFN-1 signaling intrudes in the mounting of adaptive immune responses by impairing anti-parasite responses driven by CD4T cells or exacerbating cytotoxic CD8T cell-mediated reactions leading to tissue damage.

**Figure 5:**
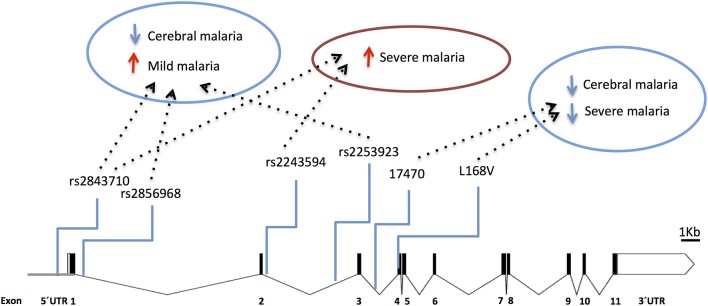
Genetic effects of IFNAR1 SNPs associated to clinical malaria. The scaled diagram of IFNAR1 exon-intron structure represents SNPs that have shown association with different malaria clinical outcomes (bar = 1 Kb). Experimental data suggest that detrimental IFNAR1 genetic effects (red arrows) could be attributed to alleles mediating decreased IFNAR1 signaling that impairs innate anti-parasite responses while beneficial effects (blue arrows) would be conferred by alleles that also decrease IFNAR1 signaling in the context of adaptive effector responses, and in particular deter cerebral immunopathology. For allele description and reported genetic effects (Supplementary Table 1).

**Figure 6:**
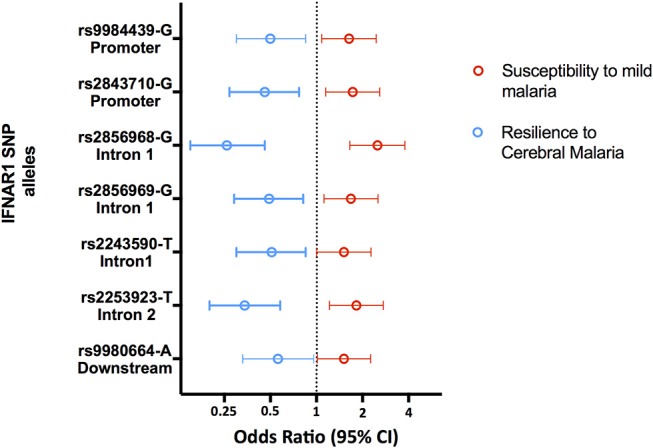
Opposing genetic effects of IFNAR1 SNP alleles in cerebral malaria and mild malaria. Genetic effects of minor frequency alleles at seven IFNAR1 SNPs in an Angolan dataset (136). Blue symbols represent the Odds Ratio and confidence intervals obtained by comparing 110 cases of CM and 129 mild malaria patients (OR< 1 corresponds to increased CM resilience). Red symbols represent the Odds Ratio and confidence intervals obtained by comparing 129 patients with mild malaria and 305 uninfected controls for the same alleles (OR > 1 corresponds to increased mild malaria susceptibility).

Accordingly, several studies identified Type 1 Interferon Receptor 1 (*IFNAR1*) gene polymorphisms associated with mild malaria, disease severity and cerebral malaria in children (136, 138–141) (Figure 5 and Supplementary Table 1). Interestingly, IFNAR1 SNP alleles that are associated with CM resilience also show to confer increased risk of mild malaria development (Figure 6). This is exemplified by the rs2843710 derived allele which showed higher frequency in mild malaria cases as compared to uninfected controls but had lower frequency in CM patients when compared to patients with mild malaria (136) (Figure 6). The duality of this pathway in human malaria is paralleled in experimental models where increased amounts of IFN-1 improve anti-parasite responses by increasing IFNAR1 signaling in early stages of infection (131) while exacerbated IFN-1/IFNAR1 signaling later in infection increases vulnerability to severe disease (136, 137).These genetic association signals would be undetectable if CM patients were directly compared with healthy individuals providing a word of caution in the design of studies investigating genetic factors that concomitantly confer susceptibility to mild malaria and resilience to severe malaria syndromes.

## Heme Oxygenase 1 (HMOX1)

Heme oxygenase-1 (HO-1) is a catabolic enzyme that cleaves heme to generate biliverdin, carbon monoxide, and ferrous iron (142). HO-1 activity has anti-inflammatory effects and has been shown to be protective against severe forms of malaria in mice (143–145). In particular, vulnerability to experimental cerebral malaria (ECM) was unearthed by the deletion of *Hmox1* (the gene encoding HO-1) in otherwise resilient mice and was abrogated by HO-1 induction or carbon monoxide administration (144). Tissue damage elicited in experimental infection is counteracted by HO-1 through reducing exposure to oxidative stress and inhibiting inflammatory action of immune cells without affecting parasite load; this represents a prototypic case of the disease tolerance phenomenon (59). In sharp contrast, modulation of *Hmox1* expression and HO-1 enzymatic activity during experimental liver stage infection revealed that HO-1 is a down-modulator of the inflammatory response against intra-hepatocytic parasite forms and acts to promote liver stage infection (146).

**Figure 7:**
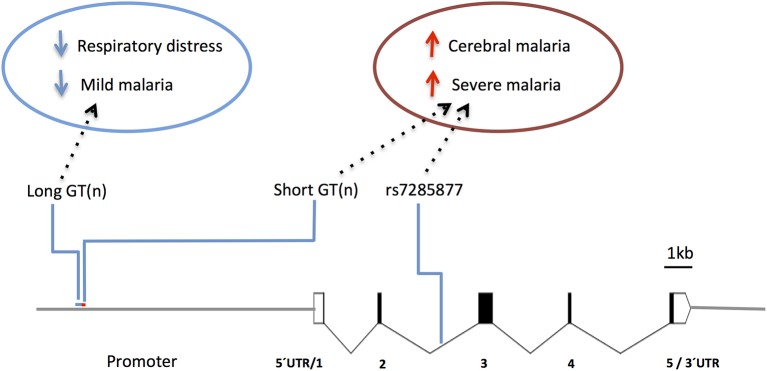
Genetic effects of *HMOX1* gene polymorphisms associated to clinical malaria. Scaled diagram of HMOX1 exon-intron structure depicting a microsatellite (GT)n repeat in the *HMOX1* gene promoter region and a malaria associated SNP in intron 2. Genetic effects of long alleles (GT)n repeat (blue) and short repeats and intronic SNP (red) have distinct association in occurrence of clinical malaria and are involved in the control of HOMX1 gene expression. For reported genetic associations (Supplementary Table 1).

Human studies focusing in the *HMOX1* gene showed that polymorphisms functionally controlling HO-1 (promoter repeat, SS) expression or activity (147) were also associated with CM vulnerability in Asia (Myanmar) (148) and Africa (Angola) (149). On the other hand, the alternative alleles in the gene promoter (repeat, L) showed protection from respiratory distress in The Gambia (150) and mild malaria in Brazil (151) (Figure 7 and Supplementary Table 1), but not in Ghana (152). It was reported that HOMX1 promoter alleles associated with disease severity in children also confer higher levels of HO-1 and enhance neutrophil respiratory burst (150). Interestingly, it was demonstrated in the mouse that expression of *Hmox1* induced by NO leads to protection from experimental cerebral malaria (114). Further research is needed to clarify how *HMOX1* gene variants act in cerebral malaria pathogenesis (150) but its tempting to speculate that genetic interaction with NOS2 functional variants may help to explain the contribution of HMOX1 to the genetic complexity of human cerebral malaria.

## Innate Sensors (TLRs and Related Genes)

Toll-like receptors (TLRs) are ancient components of the immune system that recognize pathogen associated molecular patterns (PAMPs) and are infection sensors at the onset of innate immune responses. Conceivably, TLR signaling could be involved both in resolving early infection and in heightening inflammation and immunopathology. It has been shown that binding to IE and recognition of *Plasmodium* moieties such as the glycosylphosphatidylinositol anchors (GPI) or hemozoin-DNA complexes can be mediated by several TLRs, providing sensor redundancy in the immuno-detection of blood stage parasites (48, 49, 153). A number of reports suggest that variants in different TLRs and their adaptor molecules are associated with control of parasite load (TLR9, rs187084), susceptibility to severe disease (TLR1, rs4833095), resilience to fatal disease (TIRAP S180L) and susceptibility to severe disease, including poor pregnancy outcomes (TLR4, rs4986790) (136, 154–160) (Supplementary Table 1). Together these studies convey the notion that recognition of *Plasmodium-*derived molecules by innate receptors contribute to the host response in different steps of the natural course of malaria likely by linking with pro-inflammatory responses, such as interferon production (131). Downstream effectors of TLR signaling include a variety of pro-inflammatory cytokines and chemokines such as IFN-γ, IL-6, TNF, IL-12, IFN-1, MCP-1, and IL-8 that take part in the amplification of anti-parasite responses during acute blood-stage infection (32, 161). Furthermore, it has been proposed that polymorphisms in TLRs influence circulating cytokines levels during *Plasmodium vivax* malaria (162). The association of different TLRs to malaria clinical outcomes suggests that *Plasmodium* components of diverse nature trigger genetically controlled innate responses that in turn determine the course of infection.

## CD36 and Adhesion Molecules

Several lines of evidence suggest that adhesion molecules expressed in endothelial cells are involved in pathogenesis of severe malaria by promoting cytoadhesion and possibly the sequestration of IE in microvessels (163, 164). CD36 is a scavenger receptor that was also identified as a receptor for *P. falciparum*-infected red blood cells (165). It has been noted that CD36 gene variants showing association to cerebral malaria are not associated to other severe malaria syndromes (74, 149). In particular, the low frequency allele (rs3211938; 1264G) in the exon 10 of CD36 was associated to cerebral malaria (166) and to higher malaria incidence [165) but favored resistance to severe anemia (167) (Supplementary Table 1). Individuals homozygous for this mutation which ablates CD36 protein surface expression showed reduced antibody response to malaria blood stage antigens (168, 169) suggesting that CD36 takes part in the initial steps of the adaptive immune response possibly by mediating phagocytosis of infected red blood cells. Although the role of CD36 in malaria is unsettled (164) these intriguing findings may be related with the multifunctional properties of CD36 namely as a co-receptor that together with TLR4-TLR6 heterodimers acts in the initiation of macrophage inflammatory responses triggered by microbial diacylated lipopeptides (170, 171). Nevertheless, it is possible that CD36 heterozygosity at rs3211938 interferes with the adhesion of IE to endothelial cells, a proposed cerebral malaria pathogenesis mechanism that involves other surface molecules expressed in endothelial cells, including ICAM-1, PECAM-1 and EPCR (164, 172–175).

## CD40 Ligand (CD40LG) and Adaptive Immunity Genes

CD40 ligand is expressed in the surface of activated T cells and binds to CD40 in B cells, regulating B cell proliferation, activation of antigen presentation cell, and immunoglobulin class switching. The CD40LG gene maps to chromosome X and hemizygous males or homozygous females for the minor allele at rs3092945 in the promoter region showed increased resistance to severe malaria (176) an effect that was detected in a multicentric study but was subjected to modifiers in different populations (70). This interesting finding raises the possibility that the genetic control of adaptive immune responses and in particular antibody responses could play a role in controlling parasitemia and thereby govern vulnerability to severe disease in exposed individuals. Accordingly, a recent study found that rs6682413 in the IL23R-IL12RB2 intergenic region was associated with severe malaria anemia (177), supporting observations that IL12 and IL23 pro-inflammatory effects are involved in pathogenesis of severe malaria syndromes (178, 179). In turn, Interferon gamma (IFN-γ) is a key mediator of inflammatory and immune responses induced primarily by interleukin-12 (IL-12). Besides its role in controlling early stages of infection, IFN-γ production by CD4T cells is a hallmark of Th1 polarization enhancing activation of CD8T cells, B cells, and macrophages that may lead to severe immunopathology (180). This is corroborated by genetic studies associating IFN-γ and IFNGR1 gene variants with malaria infection and cerebral malaria (140, 181).

## Prospective Remarks

The course of infection by the malaria parasite is contingent upon multiple factors including pathogenicity of parasite biotypes, transmission dynamics and host genetic make-up, that determine (im)balances between parasite expansion and host responses that in turn drive different clinical outcomes. The search for host genetic factors governing the development of clinical malaria phenotypes has attracted significant research efforts in the recent years. Under the selective pressure imposed by severe malaria, genetic variants with strong effects surfaced in endemic regions and were revealed in genes coding for proteins expressed in erythrocytes, prominently hemoglobin (74).

**Figure 8:**
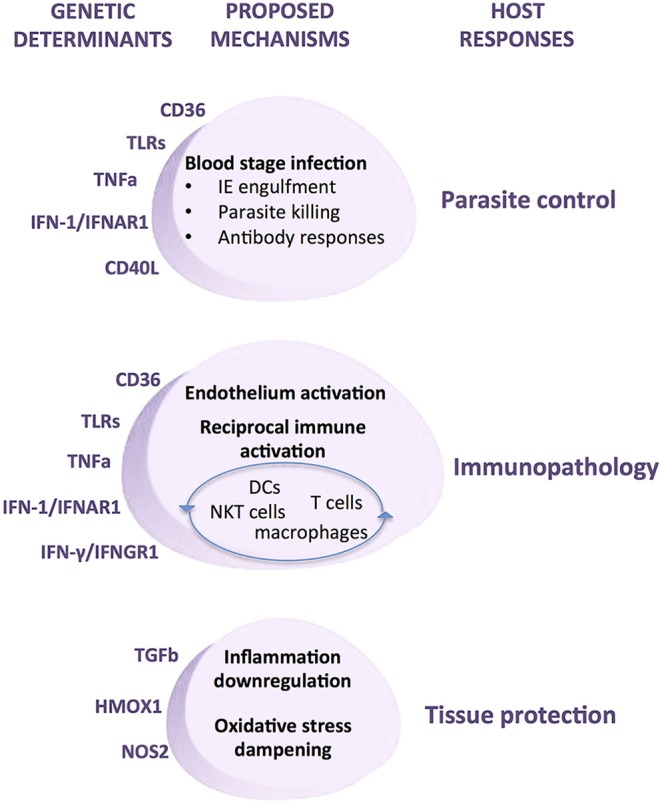
Mechanisms of genetic determination of malaria infection outcomes by inflammatory response genes. Genetic modifiers of human malaria infection have been repeatedly identified in a number of inflammatory response genes. Experimental evidence supports the involvement of these genetic determinants in multiple cellular and molecular mechanisms that underlie the host responses to malaria infection, namely control of parasite load, immunopathology, and tissue damage protection. Under this pathogenesis model specific genetic modifiers of inflammatory responsiveness may play disparate roles in the host responses to infection depending on contextual variables, including disease transmission dynamics, parasite virulence factors, infection stage, tissue-specific cues, and the overall inflammatory mellieu.

In contrast, inflammatory response genes associated with malaria show smaller genetic effects but unveiled key steps of the host response in the course of infection namely, innate recognition, phagocytosis, exacerbated inflammation, endothelial activation, and immune adaptive responses (Figure 8). Thus, innate receptors recognizing parasite moieties, including TLRs appear to be critical triggers of anti-parasite responses and together with other molecules that are also expressed in phagocytes such as CD36 highlight the central role of phagocytosis in combating infection. A number of pro-inflammatory cytokines and cytokine receptors have been identified in different association studies, including TNF and IFNAR1 but also interferon response genes and TGF beta 2 (Supplementary Table 1). These genetic associations collectively implicate amplification of the inflammatory response as a relevant determinant of infection outcomes. The role of exacerbated inflammatory responses in endothelial activation that underlie severe organ damage is further highlighted by the action of anti-inflammatory mediators such as NO and HO-1 and also by cell surface receptors promoting IE adhesion (Figure 8). Moreover, malaria genetic association with molecules that engage and polarize adaptive immune responses, including CD40L, IL4 (Supplementary Table 1), IFNGR1 (181), and TIM1 (182) underline a role for antibodies in infection resolution. Accumulation of genetic evidence revealing the involvement of inflammatory response genes in malaria provided an invaluable contribution to uncover nodes of the immune host responses that determine deleterious infection trajectories (Figure 8). The biological and physiological relevance of specific human genetic variants and their impact in disease pathogenesis is now experimentally approachable by usage of genome editing methodologies that allow precise introduction of defined mutations in malaria animal models.

The scrutiny of inflammatory genes genetic variants associated with malaria reveals a complex genetic architecture with multiple levels of genetic heterogeneity, namely:

1. Alternative alleles in one gene either favor or counteract severe disease development (e.g., HMOX1), offering direct interpretation of their genetic effects;
2. Different genetic variants within a gene promoter are associated with different severe malaria syndromes (e.g., TNF) suggesting differential gene regulation in context of different inflammatory milieus;
3. Alleles conferring both resilience to severe disease and susceptibility to asymptomatic infection/mild malaria (e.g., IFNAR1) suggesting that advantages of selecting for anti-inflammatory alleles carry fitness costs in infection susceptibility and chronic infection;
4. Regulatory variants acting prior infection or during infection map in different gene regions (e.g., NOS2), suggesting that infection status alters gene regulatory regimens.
5. Genetic variants that show disparate roles in malaria pathogenesis depending on their homo/heterozygous state (e.g., CD36).

In this scenario, evaluation of genetic effects requires that the overall allelic variation within an entire gene is taken into account. New genotyping by sequencing methodologies that generate long reads in single DNA molecules enables access to real-haplotype data across entire genes. Data on entire gene variants will allow a comprehensive analysis of their genetic effects in context of genotype combinations with other genes. These data will open new approaches to study non-allelic genetic interactions (e.g., TNF-NOS2 or NOS2-HMOX1 interactions) and to dissect the genetic architecture of inflammatory responses to malaria.

In conclusion, detailed analysis of genetic effects of inflammatory response gene variants is a key step in malaria research to motivate experimental investigations of the underlying pathogenesis mechanisms. This knowledge will be critical to identify rational adjuvant therapies to prevent fatality or undesired malaria complications and subsequent long-term sequels that represent a high burden in endemic regions.

## Author Contributions

The author confirms being the sole contributor of this work and has approved it for publication.

### Conflict of Interest Statement

The author declares that the research was conducted in the absence of any commercial or financial relationships that could be construed as a potential conflict of interest.
