# Supplementary material for: Genetics of Malaria Inflammatory Responses: A Pathogenesis Perspective
Source: Front Immunol. 2019 Jul 30;10:1771. doi: 10.3389/fimmu.2019.01771 (PMC6682681; doi:10.3389/fimmu.2019.01771)
Supplement: Supplementary file 1 [file Table_1.pdf]

Supplementary Table 1: Representative variants of inflammation-related genes reported in malaria association studies.

GENE (variant references)	Study (a)	Cases	Controls	Genetic effect	Population	OR (b)	-log10(P) (c)	notes	Ref
**Tumor Necrosis factor (TNF)**									
TNF (rs1799964, C; -1031)	C-S	severe malaria	healthy	susceptibility to severe malaria	Vietnam	1.16	1.5~2		(Dunstan et al., 2012)
	C-S	severe malaria	uninfected	susceptibility to severe malaria	India	1.76	1.5~2		(Sinha et al., 2008)
	C-S	severe malaria	non-severe	vulnerability to severe malaria	India	1.87	1.5~2		(Sinha et al., 2008)
TNF (rs1800630, C; -863)	C-S	malaria in pregnancy	uninfected pregnant	susceptibility to malaria in pregnancy	Saudi Arabia	4.64	2~3		(Nasr et al., 2014)
	C-S	severe malaria	non-severe	vulnerability to severe malaria	India	1.84	1.5~2		(Sinha et al., 2008)
TNFP-D (rs1799724, T;- 857)	C-S	cerebral malaria	mild malaria	vulnerability to cerebral malaria	Myanmar	34.5	2~3	****	(Ubalee et al., 2001)
TNF U04	C-S	cerebral malaria	mild malaria	vulnerability to cerebral malaria	Thailand	2.5	2~3	§	(Hananantachai et al., 2007)
TNF2 (rs1800629, A; -308)	C-S	cerebral malaria	uninfected	susceptibility to cerebral malaria	The Gambia	4.0	1.5~2	****	(McGuire et al., 1994)
	C-S	cerebral malaria	mild malaria	vulnerability to cerebral malaria	The Gambia	-	1.5~2	****	(McGuire et al., 1994)
	C-S	death/ neuro sequels	uninfected	susceptibility to neuro sequels/fatality	The Gambia	7.7	2~3	****	(McGuire et al., 1994)
	C-S	death/ neuro sequels	mild malaria	vulnerability to neuro sequels/fatality	The Gambia	-	2~3	****	(McGuire et al., 1994)
	C-S	severe malaria	mild malaria	vulnerability to severe malaria	Sri Lanka	3.18	2~3	****	(Wattavidanage et al., 1999)
	Long	severe malaria	-	susceptibility to re-infection	Gabon	-	1.5~2		(Meyer et al., 2002)
	Long	high parasitemia	N/A	parasitemia control	Kenya	1.1	-	****	(Aidoo et al., 2001)
TNF (rs673, A; -244)	C-S	high parasitemia	low parasitemia	parasitemia control	Congo	-	-		(Nguyen et al., 2017b)
TNF (rs361525, G; -238)	C-S	cerebral malaria	asymptomatic malaria	progression to cerebral malaria	Nigeria	5.53	2~3		(Olaniyan et al., 2016)
	C-S	cerebral malaria	mild malaria	vulnerability to cerebral malaria	Nigeria	3.69	3~4		(Olaniyan et al., 2016)
	C-S	severe malaria	mild malaria	vulnerability to severe malaria	Nigeria	2.94	2~3		(Olaniyan et al., 2016)
	C-S	severe malarial anemia	mild malaria	resilience to severe malarial anemia	The Gambia	0.52	1.5~2	****	(McGuire et al., 1999)
	Long	peak parasitemia	asymptomatic malaria	progression to clinical malaria	Burkina-Faso	-	3~4		(Flori et al., 2005)
TNF(rs3093664, A; 1034)	Long	malaria attack/peak	asymptomatic malaria	progression to clinical malaria	Burkina-Faso	-	2~3		(Flori et al., 2005)
**Nitric Oxide Synthase 2 (NOS2)**									
NOS2 (rs11080358; upstream)	C-S	asymptomatic carriers	non-carriers	resistance to infection	S. Tome	0.77	1.5~2		(de Jesus Trovoada et al., 2014)
NOS2 (CCTTT, < 11 repeats)	C-S	fatal cerebral malaria	CM survivors	vulnerability to fatal cerebral malaria	The Gambia	1.9	1.5~2		(Burgner et al., 1998)
(CCTTT, <15 repeats)	C-S	severe malaria	mild malaria	vulnerability to severe malaria	Thailand	0.41	2~3	*	(Ohashi et al., 2002)
NOS2 (-1659T)	C-S	cerebral malaria	non-malaria patients	vulnerability to CM	The Gambia	1.3	1.5~2		(Burgner et al., 2003)
NOS2 (rs8078340; upstream)	C-S	pre-erythrocytic abs	N/A	resistance to pre-erythrocytic infection	S. Tome	--	3~4		(de Jesus Trovoada et al., 2014)
	C-S	asymptomatic carriers	non-carriers	resistance to infection	S. Tome	0.82	1.5~2		(de Jesus Trovoada et al., 2014)
NOS2 (-1173 C>T)	C-S	mild malaria	healthy controls	protection from clinical malaria	Tanzania	0.12	3~4		(Hobbs et al., 2002)
	Long	severe malaria anemia	-	resistance to severe malaria anemia	Kenya	0.25	3~4		(Hobbs et al., 2002)
NOS2 (rs 1800482, -954 G>C)	Long	nr. malaria episodes	N/A	resistance to clinical malaria	Gabon	--<br>--	1.5~2<br>2~3		(Kun JF, et al., 1998)<br>(Kun et al., 2001b)
	Long	nr. malaria episodes	N/A	resistance to clinical malaria	Uganda	0.69<br>0.59	1.5~2<br>2~3		(Parikh et al., 2004)<br>(Lwanira et al., 2017)
	C-S	mild malaria	healthy controls	resistance to clinical malaria	Cameroon	0.50	2~3		(Apinjoh et al., 2014)
	Long	severe malaria	mild malaria	resilience to severe malaria	Gabon	0.67	1.5~2		(Burgner et al., 1998)

NOS2 (rs3794767; intron 2)	C-S	asymptomatic carriers	non-carriers	susceptibility to infection	S. Tome	1.53	4~5		(de Jesus Trovoada et al., 2014)
	C-S	pre-erythrocytic abs	N/A	susceptibility to infection	S. Tome	--	1.5~2		(de Jesus Trovoada et al., 2014)
NOS2 (rs16966563; exon 4)	C-S	cerebral malaria	mild malaria	resilience to cerebral malaria	Angola	0.43	3~4		(de Jesus Trovoada et al., 2014)
**Type I Interferon Receptor 1(IFNAR1)**									
IFNAR1 (rs2843710, G; -576)	C-S	cerebral malaria	mild malaria	resilience to cerebral malaria	Angola	0.56	2~3		(Ball et al., 2013b)
	C-S	mild malaria	uninfected	susceptibility to mild malaria	Angola	1.59	2~3		(Ball et al., 2013b)
	C-S	severe malaria	uninfected	susceptibility to severe malaria	India	2.0	1.5~2		(Kanchan et al., 2015)
	C-S	non-severe malaria	uninfected	susceptibility to mild malaria	India	3.03	3~4		(Kanchan et al., 2015)
	C-S	severe malaria	uninfected	susceptibility to severe malaria	Gambia, Kenya and Vietnam	1.38	3~4	**	(Khor et al., 2007)
IFNAR1rs2856968 (A/A; intron 1)	C-S	cerebral malaria	mild malaria	resilience to cerebral malaria	Angola	0.14	4~5		(Ball et al., 2013b)
	C-S	mild malaria	uninfected	susceptibility to mild malaria	Angola	1.99	4~5		(Ball et al., 2013b)
IFNAR1 rs2243594 (A, intron 2)	C-S	severe malaria	uninfected	susceptibility to severe malaria	Gambia, Kenya and Vietnam	2.67	2~3		(Khor et al., 2007)
	C-S	severe malaria	uninfected	susceptibility to severe malaria	India	2.67	4~5		(Kanchan et al., 2015)
IFNAR1 rs2253923 (T, intron 2)	C-S	cerebral malaria	mild malaria	resilience to cerebral malaria	Angola	0.48	3~4		(Ball et al., 2013b)
	C-S	mild malaria	uninfected	susceptibility to mild malaria	Angola	1.61	2~3		(Ball et al., 2013b)
IFNAR1 17470 (G/G, intron 3)	C-S	severe malaria	uninfected	resistance to severe malaria	The Gambia	0.74	1.5~2		(Aucan et al., 2003b)
	C-S	cerebral malaria	uninfected	resistance to cerebral malaria	The Gambia	0.64	2~3		(Aucan et al., 2003b)
IFNAR1 L168V (G/G, exon 4)	C-S	severe malaria	uninfected	resistance to severe malaria	The Gambia	0.69	1.5~2		(Aucan et al., 2003b)
	C-S	cerebral malaria	uninfected	resistance to cerebral malaria	The Gambia	0.76	1.5~2		(Aucan et al., 2003b)
IFNAR1 rs914142 (G,A, intron 10)	C-S	cerebral malaria	mild malaria	resilience to cerebral malaria	Nigeria	0.34	2~3		(Feintuch et al., 2018)
**Interferon response genes**									
IFN alpha 17(rs9298814, G)	C-S	non-severe malaria	uninfected	susceptibility to mild malaria	India	2.35	1.5~2		(Kanchan et al., 2015)
IFN beta 1 (rs139262191)	C-S	cerebral malaria	asymptomatic	progression to cerebral malaria	India	20.3	1.5~2	***	(Jha et al., 2013)
IRF1 (rs260638)	M-C	severe malaria	healthy	susceptibility to severe malaria	Africa/Asia	0.94	2~3		(Rockett et al., 2014)
**Heme Oxigenase 1 (HMOX1)**									
HMOX1 (promoter repeat, SS)	C-S	cerebral malaria	mild malaria	vulnerability to cerebral malaria	Myanmar	-	2~3		(Takeda et al., 2005)
	C-S	cerebral malaria	mild malaria	vulnerability to cerebral malaria	Angola	-	2~3		(Sambo et al., 2010)
HMOX1 (promoter repeat, L)	C-S	respiratory distress	mild malaria	resilience to respiratory distress	Gambia	0.15	2~3		(Walther et al., 2012)
	C-S	mild malaria	asymptomatic malaria	protection from mild malaria	Brazil	-	2~3		(Mendonça et al., 2012)
HMOX1 (rs7285877, C; intron 2)	C-S	cerebral malaria	severe non-cerebral malaria	vulnerability to cerebral malaria	Angola	1.85	1.5~2		(Sambo et al., 2010)
**TLRs and related genes**									
TLR1 (S248N)	C-S	pregnancy malaria	uninfected pregnants	susceptibility to infection in pregnancy	Ghana	2.01	1.5~2		(Hamann et al., 2010)
TLR1 (rs4833095, TT)	C-S	severe malaria	uninfected	susceptibility to severe malaria	PNG	0.52	2~3	****	(Manning et al., 2016)
TLR1 (602S)	C-S	mild malaria	asymptomatic malaria	progression to clinical malaria	Brazil	2.2	1.5~2		(Leoratti et al., 2008)
TLR4 (rs4986790; D299G)	C-S	severe malaria	uninfected	susceptibility to severe malaria	Ghana	1.5	1.5~2		(Mockenhaupt et al., 2006b)
	C-S	low birth weight	normal birth weight	vulnerability to low birth weight	Ghana	5.7	1.5~2		(Mockenhaupt et al., 2006c)
TLR4 (rs4986791; T399I)	C-S	severe malaria	uninfected	susceptibility to severe malaria	Ghana	2.6	1.5~2		(Mockenhaupt et al., 2006b)
TLR5 (rs5744105; 392Stop)	C-S	infected	non-infected	susceptibility to infection	Brazil	2.1	1.5~2		(Costa et al., 2017)
TLR6 (249S)	C-S	mild malaria	asymptomatic malaria	protection from clinical malaria	Brazil	2.0	1.5~2		(Leoratti et al., 2008)

TLR9 (rs187084, T; -1486)	C-S	low birth weight	normal birth weight	vulnerability to low birth weight	Ghana	5.7	1.5~2		(Mockenhaupt et al., 2006c)
	C-S	high parasitemia	low parasitemia	vulnerability to high parasitemia	Brazil	-	2~3		(Leoratti et al., 2008)
TLR9 (rs5743836, T; -1237)	C-S	infected	uninfected	susceptibility to infection	Brazil	1.3	1.5~2		(Leoratti et al., 2008)
	C-S	infected	uninfected	susceptibility to infection	Brazil	1.3	1.5~2		(Costa et al., 2017)
TIRAP S180L	C-S	fatal outcome	survivors	resilience against fatal outcome	India	0.31	2~3		(Panda et al., 2016)
	C-S	severe malaria	mild malaria	resilience to severe malaria	India	0.68	1.5~2		(Panda et al., 2016)
**CD36**									
CD36 (rs3211938; 1264G)***	MC	MSP2 antibodies	N/A	reduced antibody response	Africa	0.24	4~5	****	(Shelton et al., 2015)
	C-S	cerebral malaria	healthy	susceptibility to cerebral malaria	Kenya	-	1.5~2		(Aitman et al., 2000)
	C-S	cerebral malaria	healthy	resistance to severe malaria anemia	Kenya	0.62	1.5~2		(Pain et al., 2001)
	Long	malaria episodes	N/A	susceptibility to re-infection	Tanzania	-	1.5~2		(Kajeguka et al., 2012)
CD36 (rs201346212, 1439C)	M-C	severe malaria	healthy	susceptibility to severe malaria	Africa/Asia	0.67	3~4		(Rockett et al., 2014)
**CD40LG**									
CD40LG (rs3092945, C; -726)	M-C	severe malaria	healthy	resistance to severe malaria	Africa/Asia	0.85	5~6	****	(Rockett et al., 2014)
	C-S	severe malaria	healthy	resistance to severe malaria	The Gambia	0.52	2~3		(Sabeti et al., 2002)
CD40LG (rs1126535, C; +220)	C-S	severe malaria	healthy	susceptibility to severe malaria	India	2.25	1.5~2		(Purohit et al., 2017)
**IL4**									
IL4 (rs2243250, T; -589)	M-C	cerebral malaria	healthy	resistance to cerebral malaria	Africa/Asia	0.89	2~3	****	(Rockett et al., 2014)
IL4 (-524 T)	C-S	antibody response	N/A	increased antibody response	Burkina Faso	-	1.5~2		(Luoni et al., 2001)
IL4 (intron3 VNTR)	C-S	severe malaria	mild malaria	vulnerability to severe malaria	Mali	-	1.5~2		(Cabantous et al., 2009)
IL-4 TTR2	C-S	clinical malaria	healthy	Resistance to clinical malaria	India	0.55	2~3	&	(Jha et al., 2012)
**Complement Receptor 1**									
CR1 (rs17047660)	M-C	cerebral malaria	healthy	susceptibility to cerebral malaria	Africa/Asia	1.09	2~3		(Rockett et al., 2014)
CR1 (rs17047661, G; 1601G)	C-S	fatal severe malaria	severe malaria	resilience to fatal severe malaria	Kenya	0.67	2~3	****	(Opi et al., 2018)
CR1 (rs17047660, G; 1590E)	C-S	cerebral malaria	healthy	vulnerability to cerebral malaria	Kenya	1.19	1.5_2		(Opi et al., 2018)
**TGF Beta 2**									
TGFB2 (rs4846478)	C-S	cerebral malaria	severe non-cerebral malaria	vulnerability to cerebral malaria	Angola	1.70	1.5~2		(Sambo et al., 2010)

(a) Study design: C-S, Cross-sectional; Long, longitudinal; M-C, multi-centric. (b) Reported risk: Odds-Ratio. (c) Indicative significance range. N/A; not applicable.
(§) TNF U04 allele (-1031C, −863C, −857C). (&) IL4 TTR2 (-590T, -34T and intron-3 VNTR R2). (*) OR, for alternative of reported allele. (**) Dominant model. (***) Rare allele. (****) Recessive model.
